# Evaluating the impact of Marie Stopes International’s digital family planning counselling application on the uptake of long-acting and permanent methods of contraception in Vietnam and Ethiopia: a study protocol for a multi-country cluster randomised controlled trial

**DOI:** 10.1186/s13063-018-2815-0

**Published:** 2018-08-04

**Authors:** Laura A. Bates, Joseph P. Hicks, John Walley, Emily Robinson

**Affiliations:** 1 University of Leeds, Leeds, LS2 9JT UK; 2 Marie Stopes International, Monitoring & Evaluation Team, Conway Street, Fitzroy Square, London, W1T 6LP UK

**Keywords:** Family planning counselling, Contraception, mHealth, Fertility, Reproductive health, Long-acting and permanent methods, Client Centred, Digital counselling

## Abstract

**Background:**

Maintaining quality of care in family planning (FP) counselling in low-resource settings is challenging. Job aids can help providers give more accurate and client-specific advice but require a provider to use them effectively and consistently. Marie Stopes International (MSI) have designed the tablet-computer based Digital Counselling Application (DCA), which prompts structured, supportive, client-specific and unbiased FP counselling. We hypothesise that a systematic exploration of clients’ fertility intentions, medical eligibility and preferences will increase their uptake of long acting and permanent methods of contraception (LAPMs).

**Methods/design:**

We will conduct a two-armed, parallel, cluster randomised control trial across all MSI clinics (clusters) in Ethiopia (24) and Vietnam (11), randomising 18 clinics to the intervention group and 17 to the control group. Intervention providers will attend a two-day DCA-use training programme, and use DCA in their FP counselling sessions. Usual care providers will counsel clients as before. We aim to recruit 75 clients who have had FP counselling per clinic (2625 total), following them up via two telephone interviews, initially within 2 days and then at 4 months. The primary outcome is defined as the proportion of clients who report choosing a LAPM following FP counselling and will include switchers (FP counselling clients who switch from using any other FP method) and adopters (FP counselling clients who adopt any FP method having not previously been using one). We will also collect secondary outcomes at the initial follow-up (including the proportion of clients reporting being recommended a LAPM by a provider and a range of measures of client experience and satisfaction) and at the 4-month follow-up (including a range of measures of continuation rates for different FP method types). In the intervention arm, we will also conduct mixed-methods sampling to assess how providers use DCA (using an observational survey of provider–client interactions), and understand users’ experiences of receiving and giving DCA-based FP counselling (through in-depth interviews).

**Discussion:**

This trial will provide novel information on the feasibility and acceptability of health worker delivered FP counselling using DCA, with robust evidence on its effectiveness at increasing the uptake of LAPMs in low-resource settings.

**Trial registration:**

ISRCTN, ISRCTN11040557. Registered on 2 March 2017 (retrospectively registered).

**Electronic supplementary material:**

The online version of this article (10.1186/s13063-018-2815-0) contains supplementary material, which is available to authorized users.

## Background

Access to a choice of contraceptive methods is important throughout a woman’s reproductive life as her circumstances and preferences change over time [1]. A greater methods mix, or range of available family planning (FP) services, to address an unmet need is promoted by the World Health Organisation (WHO) [2, 3] and the United Nations [4].

FP service providers inform clients about available methods in different ways across the private and public sectors, ranging from group information sessions introducing all methods, to individual screening of a client’s needs and wants and supporting a client’s selection of methods. Paper-based handheld job aids, such as flipcharts, are often used as reference manuals by providers and decision-making aids for clients [5]. Information leaflets and posters, reproductive anatomy models and displays of contraceptive methods also support provider–client consultations, and the WHO eligibility wheel is also widely used in low-income countries to support providers in giving guidance on medically appropriate methods [6]. However, the application of these job aids varies across the private and public sectors, as well as between providers within the same organisation.

Maintaining quality of care in FP counselling services is vital for maximising service utilisation. Quality FP counselling requires that providers offer impartial advice on a broad mix of methods, by sharing details on side effects and effectiveness of methods [7, 8]. FP service providers must also understand their clients’ fertility intentions and address their concerns towards different methods [9]. Yet providers’ counselling can be confounded by various technical competency biases influenced by factors such as their training, cadre, time available to counsel sufficiently and demeanour [7, 10, 11]. Achieving quality FP counselling can improve client satisfaction, improve clients’ use of services, improve safe and effective use of contraceptive methods [5] and influence the likelihood of discontinuation or switching to another method [1].

Contraceptives are used by two-thirds of married or women in-union around the world, and almost 90% of contraceptive users use modern methods [4]. However, in 2015 less than half the total demand for FP to stop or limit childbearing was being met by modern methods, particularly in areas where contraceptive use is low, such as Africa, or where couples rely on traditional methods [4]. Contraceptive preferences and the promotion of methods varies by country [12]. In Africa, short-acting methods are more common whereas long-acting or permanent methods of contraception (LAPMs) are more common in Asia. These preferences are influenced by a country’s political history, access to a method mix and, in some cases, cultural preferences and provider bias [13]. In Ethiopia, the contraceptive prevalence rate is low (29%), and short-acting methods dominate the contraceptive method mix [14]. There is a skewed preference for injectable contraceptives (74%), despite community-level provision and a scaling up of long-acting methods since 2009 [14, 15]. However, use of implants is increasing, from 0.2 to 5% in 2014, and they are now the second most selected method [14]. In Vietnam, the contraceptive prevalence rate is much higher (65%), yet abortion rates are also high [16]. The two child per married couple policy of Vietnam in the 1960–1990s meant intrauterine devices (IUDs) and tubal ligation were the only modern methods promoted. However, for many women, IUDs are not medically appropriate [17] and this lack of contraceptive choice coupled with the social stigma of buying condoms and lack of knowledge of contraceptives has resulted in the highest abortion rate in Asia.

Marie Stopes International (MSI) seeks to help women choose a contraceptive method that suits their individual lifestyles and medical needs. In the past, MSI provided FP counselling training for providers that focused on the delivery of information from providers to the clients. More recently, MSI’s training has changed, placing greater emphasis on meeting the clients’ contraceptive needs and preferences. Currently, MSI providers use flipcharts to support their FP counselling sessions. However, providers typically use the flipcharts intermittently and there is substantial variation between providers’ consideration of clients’ circumstances and the suitability of methods recommend. This can result in providers offering unnecessary or an overwhelming number of options to clients, or just offering a subset of suitable options.

Therefore, given the increasingly affordable access to tablet computers, the Medical Development Team at MSI developed a tablet-computer based digital job aid, known as the Digital Counselling Application (DCA). The aim of DCA is to provide a tool that: (1) promotes interaction between providers and clients by forcing providers to ask clients a structured and systematic series of questions and (2) provides the client with a personalised list of recommended methods, based on an algorithm that uses information about the client’s situation, which the provider enters into DCA, removing the need for the provider to do this in their head (and thereby provide advice of varying quality of specificity). Women’s unmet need for modern methods, including LAPMs, is significantly associated with women’s education and empowerment, the quality of client counselling, partner discussions and exposure to media [18, 19].

In recognition of these factors, the intervention aims to support the uptake of modern methods through quality client-centred counselling. The intention is to standardise the quality of care in FP counselling to inform clients and empower them to select their preferred method. We hypothesise that DCA will offer a more client-focused counselling process, increasing providers’ consideration of clients’ circumstances and needs, reducing bias in provider recommendations, limiting discussion of all possible FP methods but expanding discussion on client-preferred methods (as ranked by DCA) designed to suit clients’ needs and preferences. This is anticipated to improve users’ counselling experience and increase clients’ readiness to consider and accept a LAPM. Our primary objective is, therefore, to evaluate the effects of DCA on LAPM uptake among clients attending FP counselling at MSI clinics in Ethiopia and Vietnam. We will also explore any effect of the DCA-led client-centred counselling on continuation rates of LAPM uptake, since providers are actively prompted to provide an upfront discussion of the side effects in advance of LAPM uptake compared to traditional counselling. The trial is also supported by a qualitative exploration of providers’ and clients’ experience of DCA, to identify where and how DCA can be improved.

## Methods/design

**Fig. 1:**
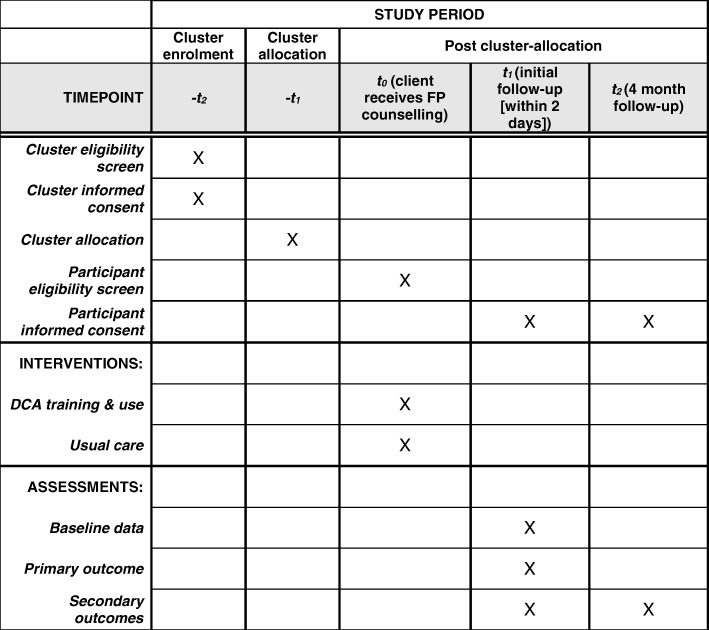
SPIRIT schedule. DCA Digital Counselling Application, FP family planning.

This protocol follows the Standard Protocol Items: Recommendations for Interventional Trials (SPIRIT) checklist (Additional file 1). The SPIRIT recommended schematic diagram detailing the schedule of enrolment, interventions and assessments is provided as Fig. 1.

### Design and study setting

We will use a parallel-group, two-arm, cluster randomised controlled trial design to evaluate whether our intervention is superior to the existing treatment (control). We will conduct the study in MSI clinics (formally known as static service delivery centres) across Ethiopia and Vietnam. All clinics in Ethiopia and Vietnam are in urban areas, and typically serve clients with higher incomes than is average for the country. In 2015, 11% of Ethiopian clients and 1% of Vietnamese clients visiting clinics lived below the poverty line of $1.25. Although MSI charges clients for FP services, they have a policy of offering a discount or waiver for clients who cannot pay. Typically, the vast majority of clients self-refer after hearing of the services offered by MSI through social networks.

Clinics are staffed by formally trained clinicians (providers), who are employed and trained by MSI, and the intervention will be delivered by MSI FP providers at the clinic level, with the clinic being the unit of randomisation. A clustered design is necessary because it will not be feasible for clinic providers to switch between delivering the intervention (which involves training) or control treatment to clients. Moreover, this design avoids the risk of contamination between different providers within clinics. In addition to the trial, we will use a structured observational survey to explore how providers are using DCA and counselling clients in the intervention arm. We will also conduct a qualitative study using in-depth interviews with providers and clients to understand their experiences of using and receiving DCA-led FP counselling, respectively, which will also help us understand if and how it can be improved.

### Participants, recruitment and consent

We will invite all MSI clinics in Ethiopia (24) and Vietnam (11) to participate in the study, via the respective MSI in-country directors. All providers will be invited to participate, and we will seek verbal informed consent from providers for their participation, but only those passing an assessment of their knowledge of the intervention at the end of the intervention training will be eligible to continue to participate.

Eligible participants will be women aged 18–49 years old who present at MSI clinics and receive FP counselling. During the recruitment phase of the trial, we will recruit participants across all participating clinics as they present consecutively for FP counselling, until clinics reach their target sample size. Clients attending MSI clinics for FP counselling will initially be asked, either by the receptionist or by the provider (if there is no receptionist), if they can be contacted by a researcher via telephone in the next 2 days who will ask them some questions about their experience of the study. Those who agree will then be telephoned in the next 2 days by a researcher for an exit interview. The researcher will then seek formal verbal informed consent from the client to conduct the exit interview and provide outcome data for the trial. They will also be asked if they can be contacted again at 4 months for a follow-up interview. Those clients who agree will then be contacted at 4 months after their FP counselling session by a researcher, again via telephone. Again they will first seek formal verbal informed consent for the interview before collecting the 4-month follow-up data. At both the initial and 4-month telephone interviews, clients who agree to be contacted but who do not answer their phone or are otherwise busy will be contacted again twice more over subsequent days, before being recorded as lost to follow-up. For clients lost to follow-up, their anonymous basic demographic data along with their choice of FP method will be collected from their clinic’s routine records (see Section 2.13 on statistical analyses for details of which patients will be included in the analyses). Clients will not receive remuneration for their time.

**Fig. 2:**
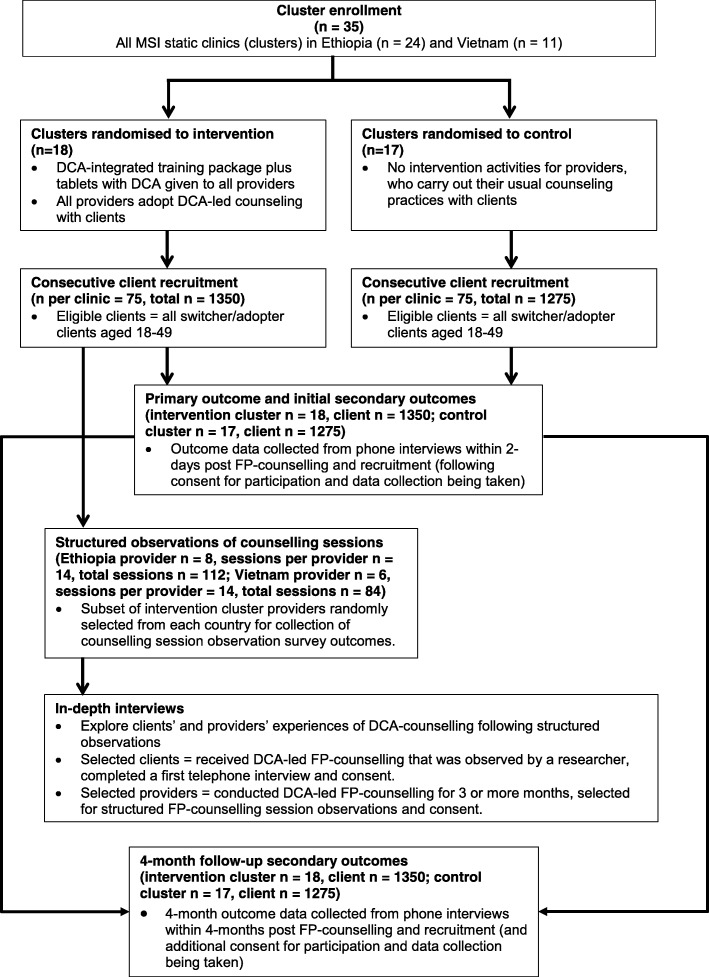
Study components flow diagram. DCA Digital Counselling Application, FP family planning, MSI Marie Stopes International

We will also conduct a structured observation survey of provider–client interactions during FP counselling sessions in intervention arm clinics. For this survey, providers will be asked to participate by a researcher, who will seek their written informed consent. The survey will then be carried out in each participating clinic over the course of a day or two, with all clients attending the clinics for FP counselling being asked to participate in the survey by the researcher, who will again seek their written informed consent. An overview of the different quantitative and qualitative components in the trial and their interlinkages is provided in Fig. 2.

### Intervention

MSI developed the intervention as part of their initiative to promote client-centred counselling on FP (particularly the provision of contraception) across all 31 countries where they operate. The intervention consists of two components: DCA and an integrated training package.

DCA is a software application that runs on an electronic tablet and, led by the provider, guides the client through a series of questions, supported by visual aids. The device and training package were developed by the Medical Development team at MSI and pilot tested in MSI clinics in South Africa to encourage feedback on its usability and content. DCA is intended for day-to-day use by trained providers during FP counselling sessions. By prompting a structured and systematic discussion and exchange of relevant information between providers and clients about the client’s preferences, it is hoped DCA will lead to more supportive, interactive and unbiased counselling sessions.

DCA prompts the user (the provider) to ask each client a series of key questions, starting with whether they are currently using a FP method. If yes, they are asked whether they are satisfied, and if no whether they have a FP method in mind or are open to exploring other methods. The clients’ answers to these questions then send them down one of three pathways. Pathway 1 is called the satisfied user check-in, and is intended for clients seeking to renew their FP method. Pathway 2 helps the client to explore all methods, and is intended to be used by contraceptive users who are not satisfied with their current method or by clients who are not currently using any method. Pathway 3 is designed to support clients with a method in mind but who are not currently using contraception.

The application gathers basic information on the client, their medical eligibility and their lifestyle, and at the end of the process provides a list of recommended methods based on the information provided, with those best fitting the clients’ responses listed at the top. The provider is then encouraged to discuss the pros and cons of the top three ranked methods with the client to explore the clients’ preferences and to help them make a decision about which contraceptive method best fits their lifestyle and needs. DCA has been designed to base its ranking of methods on clients’ fertility intentions and how effective (with regard to failure rates) the method is. The application also reflects the methods available at each centre. The application is not designed to explicitly promote the uptake of LAPM, but DCA’s intention is to offer client-centred counselling responsive to clients’ needs, which we expect to lead to an increased uptake of LAPM as more suitable and effective methods for many women’s FP needs. This means DCA is designed to prompt the provider to identify each client’s fertility intentions, medical eligibility and individual preferences, and inform each client of the possible side effects and complications of methods deemed by DCA most suitable to them. Side effects and complications are commonly left undiscussed, and so we also seek to explore if client-centred counselling and an upfront discussion of the barriers to uptake have any effect on clients’ continuation rates. Providers using DCA will discuss the most suitable methods with the client (including misconceptions, side effects and benefits) and thereby increase clients’ readiness and likelihood of accepting a suitable LAPM option (which offers greater effectiveness).

The integrated training package will consist of a 2-day programme delivered to FP providers in Ethiopia and Vietnam by MSI UK and in-country programme staff. The first day of the programme will consist of a 45-min presentation and group discussion on client-centred counselling (what it is, barriers to achieving it, how it may be different to usual counselling and its potential benefits), a 90-min introduction to DCA (including using the tablet and the application), a 90-min detailed review of the DCA content (exploring hypothetical client journeys through the application) followed by a 75-min session for hands-on practice with the application and reflection. Day two of the programme will consist of a further practice session with the application using two role plays, followed by knowledge tests and practical assessments. We will deliver the training package to all clinic providers in the intervention arm as a group workshop in Vietnam and then Ethiopia. The same training format and role plays will be used to promote consistency in providers’ interpretation of the scenarios and use of the application during those scenarios. The output of the training will involve a true/false knowledge test on contraceptive methods, and providers will be included in the trial only if they pass all questions.

Following implementation of the intervention, UK-based MSI project staff will remain in close contact with in-country MSI project staff to promote adherence to the intervention by providers in the intervention arm and to monitor the conduct of the trial.

### Control

In the control arm, clinics providers will continue to offer the usual provider–client FP counselling service. Typically, this means providers offer an overview of most or all methods before asking the client to select one. However, if providers in control arm clinics have not received MSI’s existing standard training on client-centred counselling within the last 18 months then they will receive refresher FP training. Providers in both the control and intervention arms will have access to their existing job aids, such as posters, diagrams, flipcharts, anatomy models and the WHO contraceptive wheel. Only providers in the intervention arm clinics will have access to and be given training on DCA and the tablets used to run DCA.

### Existing procedures

Aside from the intervention procedures, and the recruitment, consent and data collection processes, clinics in both arms will be allowed to operate as normal with no other changes to their day-to-day practices.

### Primary outcome

The primary outcome will be reported LAPM uptake. This is defined as the proportion of switchers and adopters who report choosing a LAPM following FP counselling (rather than a short-acting method). Switchers are clients using any FP method at the time of counselling who then change to any other FP method following counselling. Adopters are clients who are not using any FP method at the time of counselling but who decide to start using any FP method following counselling. LAPM methods include contraceptive implants, IUDs, intrauterine systems and tubal ligation, with all other FP methods considered short-acting methods. This outcome will be calculated from data collected at the initial telephone exit interview, and its accuracy is, therefore, dependent on the accuracy of clients’ responses. This primary outcome (reported LAPM uptake) is already used by MSI as a clinic performance indicator, making it feasible to collect this information. It is also a key indicator of FP practices supporting sustainable development goals 3 and 5.

### Secondary outcomes

The following secondary outcomes use data from initial client exit telephone interviews:

- Proportion of clients recommended a LAPM by their provider.
- How well clients felt providers listened to their needs, measured on a four-point Likert scale: (1) listened well, (2) listened quite well, (3) did not listen well or (4) did not listen at all well.
- The degree to which clients felt the methods recommended by providers were relevant to their needs, measured on a four-point Likert scale: (1) very relevant, (2) a bit relevant, (3) not very relevant or (4) not at all relevant.
- How well clients felt they understood the potential side effects of their chosen method, measured on a four-point Likert scale: (1) understand very well, (2) understand quite well, (3) do not understand well or (4) do not understand at all well.
- How clients felt about the amount of information given to them during counselling sessions, measured on a five-point Likert scale: (1) much too little, (2) slightly too little, (3) about right, (4) slightly too much or (5) much too much.
- How clients felt about the length of their counselling sessions, measured on a five-point Likert scale: (1) much too short, (2) slightly too short, (3) about right, (4) slightly too long or (5) much too long.
- How clients rated their experience of receiving counselling, measured on a five-point numeric scale where 1 was “bad” and 5 was “good”.

The following secondary outcomes use data from client 4-month telephone interviews:

- Proportion of adopters or switchers who chose a LAPM following their FP counselling session and reported still using the same method at 4 months.
- Proportion of adopters or switchers who chose a LAPM following their FP counselling session and who report that they plan to continue using their current method.
- Proportion of clients who chose any short-acting FP method following their FP counselling and reported still using the same method at 4 months.
- Proportion of clients who chose any short-acting FP method following their FP counselling and who report that they plan to continue using their current method.
- How clients rated their overall satisfaction with the FP method they chose following their FP counselling at 4 months, measured on a five-point numeric scale where 1 was “bad” and 5 was “good”.

### Provider observation checklist outcomes

The outcomes for the provider observation checklist consist primarily of closed-ended options to observed actions relating to a range of desirable provider behaviours, including those relating to clearly and sensitively explaining important information about the counselling session and FP methods available, and interpersonal skills and general conduct. The observation survey will also record the number of methods recommended to clients, the method type chosen by clients and the length of counselling sessions.

### Sample size

Based on logistical considerations, we assume that we can recruit 75 clients per clinic across 35 clinics during the recruitment period. Based on routine MSI clinic data, we estimate that the primary outcome of LAPM uptake in switchers and adopters is currently typically around 40%. We, therefore, estimate that we will have 80% power to detect an absolute increase in LAPM uptake from 40% to 50.5% (judged by trial researchers and MSI FP experts to be a sufficiently small, clinically important difference for these contexts) at a 5% level of statistical significance, assuming a between-cluster coefficient of variation of 0.2 (based on prior MSI routine data), with 18 clinics in the intervention arm and 17 in the control arm, and 75 clients recruited per clinic [20].

### Provider observation survey sample size

We based the sample size for our provider observation survey on being able to estimate all our proportional outcomes with a precision (95% confidence intervals) no wider than ±10 percentage points (i.e. assuming the least precisely estimable proportion of 0.5), which for an assumed design effect of 2 requires 193 observations. Logistically, we have resources to observe 14 providers, and we, therefore, plan to conduct 196 observations, split between eight providers in Ethiopia and six in Vietnam (14 observations per provider).

### Randomisation, allocation concealment and masking

For the main trial, all 35 clinics across Ethiopia and Vietnam will be randomised by MSI (ER) simultaneously via a computer program to either the intervention or control arm in a restricted 18:17 (intervention : control) ratio, following recruitment of providers in clinics and after obtaining provider consent. It will, therefore, be possible to conceal treatment arm allocation from providers and clients, but during the trial it will not be possible to blind (mask) either providers or clients to treatment allocation, given the explicit nature of the intervention components (e.g. following treatment allocation, intervention providers will be trained, provided with tablets and use DCA to counsel clients, and clients will be taken through the DCA counselling process). However, data collectors (telephone interviewers) will be blinded to the allocation of clients. For the provider observation survey, we will subsequently select, via simple random selection, eight providers in Ethiopia and six in Vietnam, from all participating clinics.

### Quantitative data collection and management

As MSI client data currently collected by clinics are not available for research, we prepared data collection tools to collect the required outcome data. There will be three data collection processes, all conducted by researchers. First, to collect data on our quantitative primary and secondary outcomes, as well as a range of relevant socio-demographic covariate data, researchers will conduct initial client exit telephone interviews using a data collection form. Second, researchers will also collect additional client outcome data 4 months after their FP counselling sessions on related secondary outcomes via further telephone interviews using another data collection form. Third, researchers will record outcomes from the structured provider observation survey via a structured observational checklist tool.

Data management will initially be the responsibility of the in-country study leaders. For each data collection process, an SPSS database will be created with consistent coding and design, and this will be duplicated for use across countries. The databases will then be collated and merged by the study statistician, and checked for quality, accuracy and completeness using standard data validation methods, such as range and logic checks. Errors, anomalous values and missing values will then be explored along with in-country MSI staff against original data forms, to clean and correct the databases where possible before the database is locked. No personally identifying data will be collected in any data collection process, and so all stored client data will be anonymous. Consent forms will, however, be linkable to client data in the databases via a generic ID, and so will be stored in secure locked cabinets. Given the nature of the intervention and the very low risk of harms, it was decided not to have a data monitoring committee for the trial.

Both MSI (ER) and the University of Leeds (JW, JPH and LAB) will have full access to the final trial and associated study datasets, although only the University of Leeds will be involved in analysing the data.

### Statistical analyses

We will use methods suitable for cluster trials with relatively few clusters, analysing cluster-level summary measures (proportions) of all binary outcomes to account for between-cluster variation, and reporting crude and covariate-adjusted results [21]. We first will convert all Likert and numeric scale outcomes into binary scale outcomes. As laid out above, the four-point Likert scale outcomes will be dichotomised by combining the first two categories and then the final two categories. The two 5-point Likert scale outcomes will be dichotomised by combining the first two categories and then the final three categories. The five-point numeric scale outcomes will be dichotomised by combining the first three categories and then the final two categories. For all outcomes, we will first estimate the crude intervention effect as the difference between the mean intervention arm and mean control arm cluster-level outcome proportions (i.e. a risk difference), and we will estimate the 95% confidence intervals and *p* value associated with this effect size estimate using an independent *t*-test.

We will then estimate covariate-adjusted results using a two-stage method [21]. First, using a logistic regression model, we will model the effects of our covariates of interest on the individual-level binary outcomes, while excluding the effect of treatment. We will then use the individual-level model predicted values and the individual-level observed values for each outcome to calculate covariate-adjusted cluster-level difference residuals. Lastly, we will use these values in place of cluster-level summary outcome measures to estimate covariate-adjusted risk differences, and again use *t*-tests to estimate the associated 95% confidence intervals and *p* values. We will adjust outcomes for covariates as shown in Table 1. In addition, using the methods outlined in Hayes & Moulton [21] for analysing cluster-level effect modification, we will assess whether the treatment effect differs for the primary outcome between the following subgroups: Ethiopia vs Vietnam and switchers vs adopters.

**Table 1:** Planned covariate adjustment for covariate-adjusted analyses

Outcome	Covariates adjusted for
Primary (LAPM uptake among FP switchers and adopters)	1–8
Proportion of clients recommended a LAPM by their provider	1–8
How well clients felt providers listened to their needs	1–8
How relevant clients felt the method(s) recommended by providers were to their needs	1–8
How well clients felt they understood the potential side effects of their chosen method	1–3, 5–6, 8
How clients felt about the amount of information given to them during counselling sessions	1–8
How clients felt about the length of their counselling sessions	1–8
How clients rated their experience of receiving counselling	1–7
Proportion of adopters or switchers who chose a LAPM following their FP counselling session and reported still using the same method at 4 months	1–9
Proportion of adopters or switchers who chose a LAPM following their FP counselling session and who report that they plan to continue using their current method	1–9
Proportion of clients who chose any short-acting FP method following their FP counselling and reported still using the same method at 4 months	1–9
Proportion of clients who chose any short-acting FP method following their FP counselling and who report that they plan to continue using their current method	1–9
How clients rated their overall satisfaction with the FP method they received following their FP counselling at 4 months	1–8

*FP* family planning, *LAPM* long-acting or permanent method of contraception, *MSI* Marie Stopes International

Covariates: (1) country (Ethiopia or Vietnam), (2) client age, (3) client education level (none, primary, secondary, college or higher), (4) marital status (single or no regular partner, regular partner, married, divorced or separated, refused to say), (5) FP intentions (trying to become pregnant now or within 1 year, aiming to become pregnant within 2+ years, do not want any or anymore children or do not know), (6) current FP usage (currently use LAPM, currently use short-acting or natural method or unsure what method, not currently using any FP method or have never used any FP method), (7) reason for visiting MSI clinic (seeking FP health care, seeking abortion health care or seeking other health care), (8) overall initial rating of FP counselling experience (1–5, where 1 is “bad” and 5 is “good”), (9) overall rating at 4 months of satisfaction with the method received (1–5, where 1 is “bad” and 5 is “good”)

We will analyse client data based on the original allocation of the clinic that they attended, but we will also conduct sensitivity analyses where we will analyse client data based on how the clinic they attended actually participated in the study (i.e. as either an intervention or control clinic). Prior to the analyses, we will assess the level and pattern of any missingness in outcome or covariate data and investigate the likely causes of missingness. We will conduct complete case analyses of outcomes unless these investigations indicate there is likely to be substantial bias introduced, in which case we will use multilevel multiple imputation methods of analysis [22]. We will make our primary inferences about the statistical significance of the results via formal hypothesis testing. Statistical significance will be at the 5% level based on the (two-sided) *p* values estimated for all outcomes obtained from the covariate-adjusted analyses, as these are likely to have more power and reduced bias (when estimating the treatment effect) than the crude analyses, which will help us to understand how any imbalances in covariates between treatment arms influence the treatment effect [23]. We will evaluate clinical significance based on the effect size estimates and their 95% confidence intervals, with an increase of 10.5 percentage points or more in the primary outcome seen as clinically significant. We will not adjust secondary outcomes for multiple testing. No interim analyses are planned. We will specify our planned analyses in a statistical analysis plan prior to analysis, which will be published with the results.

We will analyse the observation checklist outcomes using survey methods appropriate for the clustered design of the study [24], via the *survey* [25] package in the statistical software R. We will primarily analyse survey checklist outcomes descriptively, either as percentages (categorical outcomes) or as means (continuous outcomes), making inferences about the plausible range of true values for outcomes based on their 95% confidence intervals. We will calculate outcome results from the pooled data and for each country separately, exploring differences in outcomes between countries based on outcome point estimates and 95% confidence intervals.

### Qualitative data collection

In-depth interviews will be conducted with a subsample of clients who received FP counselling with the application, whose counselling session was observed and who completed a first telephone interview and consented to being contacted in the future. Purposive sampling will seek to select individuals who received different methods. Clients recruited to the telephone interview will be called again by a researcher, who will seek verbal consent to meet with them and record an in-depth interview. Informed consent will be taken when the client and researcher meet face to face, using a digital recorder to record the client’s consent to be interviewed.

In-depth interviews will be conducted with a subsample of providers who have been trained to use DCA within the intervention arm, who were observed while providing counselling and who are available to undertake an in-depth interview. Purposive convenience sampling will be used to recruit providers, to ensure that providers of all cadres are interviewed. The researcher will seek verbal consent to meet with one provider per clinic and record an in-depth interview. Informed consent will be taken when the provider and researcher meet face to face, using a digital recorder to record the provider’s consent to be interviewed.

A topic guide will be prepared by LAB to structure the interviews. These topic guides will be reviewed in advance by the research team, pre-tested prior to implementation and revised accordingly. With clients, we will explore their experiences and attitudes to FP prior to the counselling session, their experience of being counselled with a provider led by DCA, their acceptance of the FP options recommended by DCA, and their reflection on any changed attitudes to LAPM. With providers, we will explore their attitudes towards good and bad counselling traits, their experience of and response to the training, the acceptability and feasibility of DCA in practice, the perceived impact of DCA on their FP counselling, and discussions around how they use it.

In-depth interviews with clients will take place within 1 month of being counselled with DCA. In-depth interviews with providers will take place after they have used DCA for 3 months. Research assistants fluent in the local language and with qualitative data collection experience will be recruited to the study to collect all telephone data. They will be given training on the study rationale, obtaining consent, confidentiality, use of data collection tools and data protection. These researchers will be supervised by their lead in-country investigator.

### Qualitative analysis and interpretation

Qualitative data will be audio-recorded and transcribed by the in-country researchers. A sample of the original transcripts will be checked by the in-country lead for quality control. All identifying information will be removed from the transcript prior to review and analysis by LAB.

In-depth interviews with clients will be analysed by thematic content analysis, using the analysis software NVivo. The framework approach [26] will be applied to help organise and compare the data. The framework approach is a systematic method that appreciates the iterative nature of qualitative data analysis. Analysis will follow four key stages: familiarisation, constructing a thematic framework, indexing and mapping, and interpretation. A thematic framework will be derived from the research objectives and emergent themes, under which the data will be organised, analysed and interpreted. We will aim to understand the acceptability to providers and clients, and the feasibility (e.g. time taken to counsel) of using DCA during FP counselling. We will also identify a set of pragmatic recommendations to improve DCA, training on DCA and the use of DCA in practice, if such changes appear necessary.

### Dissemination

Following completion of the study (after all 4-month follow-up data have been collected), we will publish the results of the trial in a peer-reviewed journal and present them at relevant international conferences. We will also report the results separately to the funder for their internal use.

## Discussion

This trial will provide evidence on the effectiveness, feasibility and acceptability of using a digital application to structure FP consultations with clients and its associated impact on LAPM. Here we will identify the merit of a digital application that aims to standardise and support providers’ interactions with clients, yet remains flexible enough to accommodate the life experiences and medical suitability of clients visiting both quiet and busy centres. MSI Ethiopia and Vietnam recognise the need for FP, which empowers women, men and young people to achieve their sexual and reproductive health rights, and reduces maternal mortality and morbidity. The trial will inform how the use of DCA may impact their daily counselling and method mix uptake.

This trial supports MSI’s commitment to provide access to quality FP counselling to women, including empowering women to choose a FP method that best suits their needs. This goal spans across MSI’s 37 country programmes, where knowledge and acceptance of all modern methods and provider-led counselling is highly varied. The trial will, therefore, contribute to the currently very limited evidence on the roles that digital job aids may play in improving the quality of counselling provided across FP clinics (both MSI’s and other organisations’ clinics). The findings of the study will be used by MSI to assess the effectiveness of DCA in improving FP outcomes and client experience. Depending on the results of the trial, the application will be considered for use in other MSI country programme centres, and it may be revised to incorporate the learning observed. The outcomes (opportunities and challenges) of the trial are also likely to be useful to governments and non-governmental organisations considering using digital job aids in FP counselling in similar health-care settings.

### Trial status

Recruitment to the trial started in Ethiopia and Vietnam in January 2017, and was ongoing at the time of submission.

## Additional file

Additional file 1: SPIRIT checklist. Completed SPIRIT checklist for this protocol. (DOC 120 kb)

## Abbreviations

DCA: Digital Counselling Application
FP: Family planning
IUD: Intrauterine device
LAPM: Long-acting and permanent method of contraception
MSI: Marie Stopes International
WHO: World Health Organisation
