# Correction to: Evaluating the impact of Marie Stopes International’s digital family planning counselling application on the uptake of long-acting and permanent methods of contraception in Vietnam and Ethiopia: a study protocol for a multi-country cluster randomised controlled trial

**DOI:** 10.1186/s13063-018-2966-z

**Published:** 2018-10-16

**Authors:** Laura A. Bates, Joseph P. Hicks, John Walley, Emily Robinson

**Affiliations:** 1 University of Leeds, Leeds, LS2 9JT UK; 2 Marie Stopes International, Monitoring & Evaluation Team, Conway Street, Fitzroy Square, London, W1T 6LP UK

## Correction

Following publication of the original article [1], the authors requested a correction to be made, indicating L. Bates as the first author only. There is no joint first authorship.
